# Deciphering insights into commercial Myrrh species authenticity from the *psb*A-*trsn*H genetic region

**DOI:** 10.1371/journal.pone.0320731

**Published:** 2025-04-07

**Authors:** Changhong Cao, Wenting Zhong, Feng Gao, Ying Zhang, Zhiguo Ma, Hui Cao, Menghua Wu

**Affiliations:** 1 Lingnan Traditional Chinese Medicine Research Centre, School of Pharmaceutical Sciences, Jinan University, Guangzhou, Guangdong, China; 2 Lingnan Resource Sub-center, National Engineering Research Center for Modernization of Traditional Chinese Medicine, Guangzhou, Guangdong, China; 3 Cao Hui National Inheritance Workshop of Famous Elderly Chinese Medicine Experts, Guangzhou, Guangdong, China; Saint Xavier's College, INDIA

## Abstract

**Objective:**

The genetic analysis, particularly focusing on the *psb*A-*trn*H region, aims to tackle the challenges linked to myrrh identification and improve quality control in medicinal and aromatic plant sectors. This process reveals the genetic diversity inherent in myrrh species, identifies adulterants, and assesses consistency with pharmacopoeia-designated species.

**Methods:**

A meticulous investigation was conducted, involving twenty-five myrrh samples sourced from diverse origins and one adulterant sample. The methodology encompassed precise execution of DNA extraction, PCR amplification targeting the *psb*A-*trn*H region, sequencing, and subsequent data analysis. Additionally, the integration of GenBank data was employed to enrich the genetic analysis.

**Results:**

The *psb*A-*trn*H region demonstrated 100% amplification efficiency across all myrrh samples, accurately identifying three distinct species—*Commiphora gileadensis*, *Commiphora myrrha*, and *Commiphora edulis*. Only 8% of samples aligned with pharmacopoeia-specified species, revealing a significant misalignment. The identified adulterant, *Liquidambar formosana*, underscored the efficacy of the genetic approach. Genetic distances and haplotype analysis offered insights into myrrh species diversity. Intraspecific and interspecific distances highlighted the discriminatory potential of the *psb*A-*trn*H region. A phylogenetic tree illustrated distinct genetic clusters among *Commiphora* species and *Liquidambar formosana*.

**Conclusions:**

It affirms the robustness of the *psb*A-*trn*H region for authenticating myrrh and emphasizes the necessity of adapting pharmacopoeial standards to accurately mirror genetic diversity. An avenue for exploring therapeutic variations within myrrh species and advocates collaboration among researchers, regulatory agencies, and industry stakeholders to fortify comprehensive quality management measures within the context of agronomy-focused herbal products.

## Introduction

Myrrh, a resin prized for over 4,000 years, has long been recognized for its medicinal properties in the Middle East and Mediterranean regions [1]. Primarily sourced from the genus *Commiphora*, including the economically significant *Commiphora myrrha*, myrrh grows in eastern and southern Africa, Madagascar, and extends to regions like Iran, Pakistan, India, Sri Lanka, and Brazil [2]. This resin is highly valued for its applications across spices, cosmetics, food, pharmaceuticals, and health products [3–6]. Chemically, myrrh contains monoterpenes, sesquiterpenes, triterpenes, steroids, and lignans, contributing to its pharmacological properties, including antipyretic, anti-inflammatory, analgesic, and neuroprotective effects, with minimal toxicity [7–13]. Historically, it has been revered globally under various names such as “Arabian myrrh” and “murr” [2].

Once revered akin to gold, myrrh’s journey from a holy oil mentioned in the Holy Bible to its recognition for therapeutic benefits unfolded gradually, leaving an indelible mark [14]. The surge in studies exploring its applications in traditional Chinese medicine and herbal medicine reflects growing interest, fueled by investments in ethnomedicine and related health industries [15].In the 2020 Chinese Pharmacopoeia and the 11th European Pharmacopoeia, *Commiphora myrrha* and *Commiphora molmol* are accepted species for myrrh. The 2024 NF42 version of the United States Pharmacopeia includes *Commiphora myrrha* (syn. *Commiphora molmol*) or other *Commiphora* species, highlighting regional differences in standards (Table 1). With China as a major myrrh importer, quality and adulteration issues, including mixing with substances like rosin and sand, pose challenges to pharmaceutical safety [16]. The demand for reliable authentication methods has intensified, as price increases exacerbate adulteration risks. Recently, genetic methods, particularly DNA barcoding through the *psb*A-*trn*H region, have been introduced as effective tools to authenticate *Commiphora* species, even in mixed or processed samples [16–19]. These advancements ensure myrrh’s quality and authenticity in its diverse applications, preserving its value in traditional and modern medicine.

**Table 1:** Myrrh origin profile in different pharmacopoeias.

No.	Edition	Pharmacopoeia	Species Included
1	2020 version	Pharmacopoeia of the People’s Republic of China	*Commiphora myrrha* or *Commiphora molmol*
2	11th version	European Pharmacopoeia (EP)	*Commiphora myrrha* (Nees) Engl.(syn. *Commiphora molmol* (Engl.) Engl.ex Tschirch) and/or other species of *Commiphora*.
3	2024-NF42 version	United States Pharmacopeia (USP)	*Commiphora myrrha* (Nees) Engl.(syn. *Commiphora molmol* (Engl.) Engl.ex Tschirch) and/or other species of *Commiphora*.

## Method

### Sample sources

**Fig 1:**
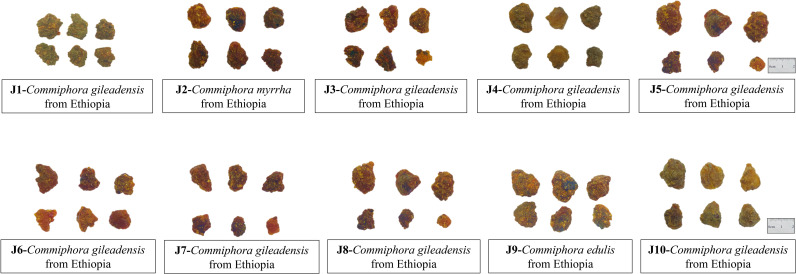
Appearance of myrrh samples labeled J1-J9.

**Fig 2:**
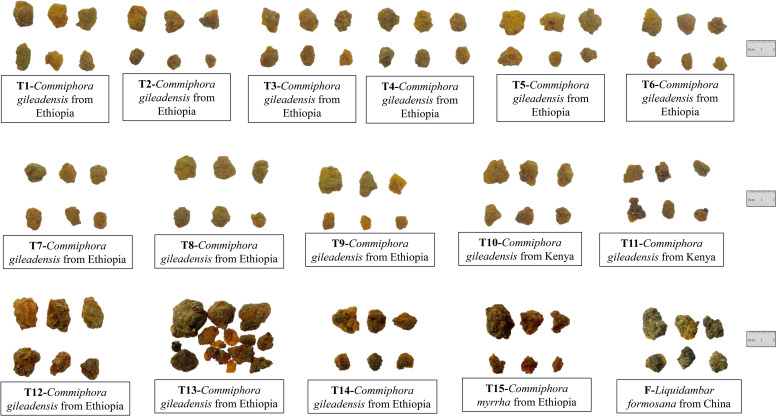
Appearance of myrrh samples labeled T1-T15&F.

To facilitate a comprehensive genetic analysis, a total of myrrh samples labeled J1-J10(Commodity name: Gum Opopozx) in Fig 1 and T1-T15(Commodity name:Gum myrrh) in Fig 2 were meticulously sourced from the Guangzhou Customs Districts in the People’s Republic of China(P.R.C). Additionally, a distinct sample of *Liquidambar formosana*, denoted as F, was collected from Huolu Mountain Forest Park in Guangzhou, China, originating from a different country of origin, thus adding diversity to our study. To ensure the accuracy, all samples underwent meticulous identification conducted by the authors (Prof. Wu and Prof. Cao), affirming their belonging to the genus Liquidambar. Further identification efforts are needed to determine specific species and genetic origins.

Following the guidelines of the Pharmacopoeia of the People’s Republic of China, our curated samples adhere to the generic species of myrrh, *Commiphora myrrha* or *Commiphora molmol* [20]. These samples have been diligently submitted to GenBank to broaden scientific inquiry and encourage collaborative research. To enhance the diversity of our genetic analysis, a distinct sequence of myrrh from the genus *Commiphora* has been downloaded from GenBank. Additionally, an outgroup represented by *Bursera malacophylla* has been included in the GenBank repository [21]. Detailed information on each sample, vital for understanding their unique genetic characteristics, is meticulously presented in Table 2, providing a solid foundation for comprehensive genetic exploration.

**Table 2:** Details of collected samples.

No.	Code Number	Country of origin	Collection sources
1	J1	Ethiopia	Guangzhou Customs Districts, P.R.C
2	J2	Ethiopia	Guangzhou Customs Districts, P.R.C
3	J3	Ethiopia	Guangzhou Customs Districts, P.R.C
4	J4	Ethiopia	Guangzhou Customs Districts, P.R.C
5	J5	Ethiopia	Guangzhou Customs Districts, P.R.C
6	J6	Ethiopia	Guangzhou Customs Districts, P.R.C
7	J7	Ethiopia	Guangzhou Customs Districts, P.R.C
8	J8	Ethiopia	Guangzhou Customs Districts, P.R.C
9	J9	Ethiopia	Guangzhou Customs Districts, P.R.C
10	J10	Ethiopia	Guangzhou Customs Districts, P.R.C
11	T1	Ethiopia	Guangzhou Customs Districts, P.R.C
12	T2	Ethiopia	Guangzhou Customs Districts, P.R.C
13	T3	Ethiopia	Guangzhou Customs Districts, P.R.C
14	T4	Ethiopia	Guangzhou Customs Districts, P.R.C
15	T5	Ethiopia	Guangzhou Customs Districts, P.R.C
16	T6	Ethiopia	Guangzhou Customs Districts, P.R.C
17	T7	Ethiopia	Guangzhou Customs Districts, P.R.C
18	T8	Ethiopia	Guangzhou Customs Districts, P.R.C
19	T9	Ethiopia	Guangzhou Customs Districts, P.R.C
20	T10	Kenya	Guangzhou Customs Districts, P.R.C
21	T11	Kenya	Guangzhou Customs Districts, P.R.C
22	T12	Ethiopia	Guangzhou Customs Districts, P.R.C
23	T13	Ethiopia	Guangzhou Customs Districts, P.R.C
24	T14	Ethiopia	Guangzhou Customs Districts, P.R.C
25	T15	Ethiopia	Guangzhou Customs Districts, P.R.C
26	F	China	Huolu Mountain Forest Park, Guangzhou, P.R.C

#### DNA extraction

To extract total genomic DNA, the DNA extraction method was adapted from Li et al [22,23]. Initially, residual bark was carefully removed from the resin [7], which was then stripped off and weighed at approximately 50 mg. A clean blade was used to scrape away any excess resin. Subsequently, the samples underwent dual sterilization: first soaking in isopropyl alcohol for 5 minutes to remove any remaining resin, followed by a 5-minute immersion in 75% ethanol to ensure sterility. Once dried, the prepared samples were dissected into smaller fragments using sterilized scissors and placed into 2 ml centrifuge tubes with grinding beads to aid in processing. To ensure thorough homogenization, the samples underwent a 6-minute grinding cycle at 70 Hz using a fully automated sample tissue grinder from Shanghai Jingxin Experimental Technology, Shanghai, China.

#### PCR Amplification and sequencing

**Fig 3:**
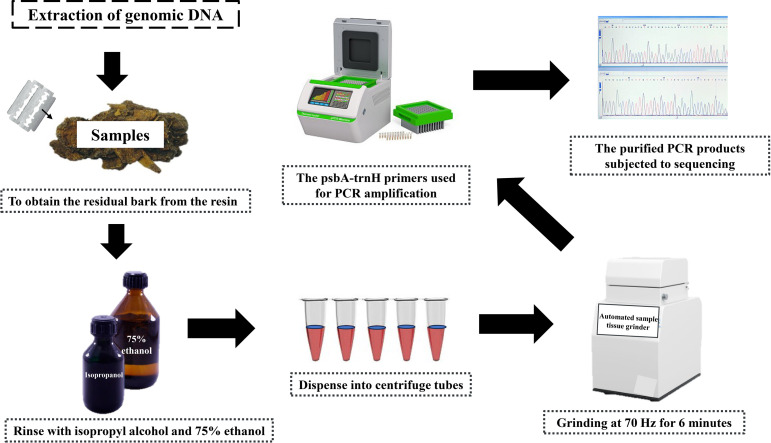
Sample collection and taxonomic identification process.

During the molecular amplification phase [24,25], the *psb*A-*trn*H region was specifically targeted using primer pairs *psb*AF (5’-GTTATGCATGAACGTAATGCTC-3’)/*trn*HR(5’-CGCGCATGGTGGATTCACAATCC-3’) (10 µ M). Polymerase Chain Reaction (PCR) was executed in a 50 μL system, incorporating approximately 10 μL of genomic DNA, 2 μL each of the forward and reverse primers, 25 μL of 2 ×  Phanta Max Master Mix (Dye Plus), and the volume was meticulously adjusted to 50 μL with sterile water. To confirm successful PCR amplification [26], the resulting products were visualized on an agarose gel. Electrophoresis was conducted in 1 ×  TBE for 20 minutes at a constant pressure of 120 V, allowing detailed examination of the amplified DNA fragments. Subsequently, purified PCR products underwent sequencing on a 3730 XL sequencer, manufactured by Applied Biosystems in Guangzhou, China. For a visual representation of the entire process, refer to Fig 3, illustrates the steps from sample preparation to sequencing.

#### Data analysis

The data analysis process unfolded systematically to ensure the robustness of our findings. Using Geneious 9.0.2 [27], we reconciled sequencing peak diagrams and assembled sequences to ensure data accuracy and integrity. This software effectively managed the complex datasets generated through our sequencing efforts. To delineate the *psb*A-*trn*H gene spacing boundaries precisely [28], we utilized annotations from similar sequences in GenBank, establishing clear demarcations for subsequent analyses. Aligning all sequences was the next focus, achieved using the MUSCLE option within Geneious 9.0.2, facilitating accurate comparisons and comprehensive insights into genetic relationships within the myrrh samples. For further exploration of genetic relatedness and confidence in our findings, a Neighbour-Joining (NJ) tree [29] was constructed using MEGA-X, provided by the Center for Evolutionary Medicine and Informatics in Tempe, AZ, USA. Bootstrapped resampling with 1000 replicates added statistical rigor, strengthening the reliability of our phylogenetic analyses. Genetic distances, calculated using the Kimura 2-parameter (K2P) model [30], provided nuanced insights into intra- and interspecific variations within our dataset. Exploring DNA barcoding gaps illuminated distribution patterns of intraspecific versus interspecific variation, enhancing our understanding of the genetic landscape.

## Results

### Characterisation of traits

Referring to Pharmacopoeia of the People’s Republic of China [17], the samples J1-J10 and T1-T15 are irregular granular masses of varying sizes, with larger ones reaching diameters of up to 6 cm or more. The texture is hard and brittle, with a crushed surface lacking smoothness and shine. They possess a distinct aroma and a bitter, slightly pungent taste. However, there are variations in color, likely influenced by different climates and origins. Some surfaces appear light yellow, yellowish-green, yellowish-brown, or reddish-brown, while certain translucent parts exhibit a brownish-black hue, often covered with yellow dust. Sample F consists of irregular lumps, ranging from light yellow to yellowish-brown, and may be translucent or opaque. The texture is hard and brittle, the crushed surface lacks smoothness and shine.

### Analysis of DNA extraction and PCR amplification

Delving deeper into the intricacies of our genetic analysis, the evaluation of DNA barcodes emerged as a critical component of our study. This involved a thorough examination of samples extracted and amplified using the *psb*A-*trn*H primer, a key genetic marker. Notably, the amplification process demonstrated flawless efficiency, achieving 100% success across all 25 myrrh samples and one *Liquidambar formosana* sample. The next step entailed sequencing all PCR products, with an emphasis on obtaining duplicate sequences of high quality. This was crucial for ensuring the reliability and consistency of our genetic data, thereby establishing a solid foundation for subsequent analyses. The results of this meticulous process are detailed in Tables 3 and 4, which encapsulate the nuances of the DNA barcodes within the sample set.

**Table 3:** Details of collected samples for genetic analysis.

Serial Number	Primer	Species*	GenBank no.
**J1**	*psb*A-*trn*H	*Commiphora gileadensis*	PP893230
**J2**	*psb*A-*trn*H	*Commiphora gileadensis*	PP893230
**J3**	*psb*A-*trn*H	*Commiphora gileadensis*	PP893230
**J4**	*psb*A-*trn*H	*Commiphora gileadensis*	PP893230
**J5**	*psb*A-*trn*H	*Commiphora gileadensis*	PP893230
**J6**	*psb*A-*trn*H	*Commiphora gileadensis*	PP893230
**J7**	*psb*A-*trn*H	*Commiphora gileadensis*	PP893230
**J8**	*psb*A-*trn*H	*Commiphora gileadensis*	PP893230
**J9**	*psb*A-*trn*H	*Commiphora edulis*	PP893232
**J10**	*psb*A-*trn*H	*Commiphora gileadensis*	PP893230
**T1**	*psb*A-*trn*H	*Commiphora gileadensis*	PP893230
**T2**	*psb*A-*trn*H	*Commiphora myrrha*	PP893231
**T3**	*psb*A-*trn*H	*Commiphora gileadensis*	PP893230
**T4**	*psb*A-*trn*H	*Commiphora gileadensis*	PP893230
**T5**	*psb*A-*trn*H	*Commiphora gileadensis*	PP893230
**T6**	*psb*A-*trn*H	*Commiphora gileadensis*	PP893230
**T7**	*psb*A-*trn*H	*Commiphora gileadensis*	PP893230
**T8**	*psb*A-*trn*H	*Commiphora gileadensis*	PP893230
**T9**	*psb*A-*trn*H	*Commiphora gileadensis*	PP893230
**T10**	*psb*A-*trn*H	*Commiphora gileadensis*	PP893230
**T11**	*psb*A-*trn*H	*Commiphora gileadensis*	PP893230
**T12**	*psb*A-*trn*H	*Commiphora gileadensis*	PP893230
**T13**	*psb*A-*trn*H	*Commiphora gileadensis*	PP893230
**T14**	*psb*A-*trn*H	*Commiphora gileadensis*	PP893230
**T15**	*psb*A-*trn*H	*Commiphora myrrha*	PP893228
**F**	*psb*A-*trn*H	*Liquidambar formosana*	PP893229

**Table 4:** Sample characteristics of the DNA barcodes evaluated.

DNA region	*psb*A-*trn*H
Number of individuals	25
Number of species	3
PCR/sequencing success (%)	100/100
Amplified sequence length (bp)	504-538
Aligned sequence length (bp)	427-437
Average GC content (%)	28.8-29.7
Variable sites	15
Haplotypes	4
Intraspecific distance range (mean)	0-0.0023 (0.0007)
Interspecific distance range (mean)	0.0083-0.0587 (0.0387)

### Identification of Myrrh and mixed counterfeit samples

A thorough BLAST analysis of our genetic data on GenBank revealed a broad diversity within the myrrh species. Among the 25 myrrh samples, 22 were identified as *Commiphora gileadensis*, 2 as *Commiphora myrrha*, and 1 as *Commiphora edulis*, with all of these sequences now uploaded to GenBank. This genetic variability within the genus *Commiphora* highlights the complexity of myrrh’s genetic composition. Such discrepancies suggest that many myrrh products may not conform to established medicinal standards, which could impact their therapeutic efficacy and safety. This emphasizes the need for more precise identification methods to ensure compliance with the Pharmacopoeia of the People’s Republic of China and to preserve the integrity of myrrh used in medicinal contexts.

**Fig 4:**
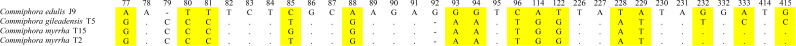
Sequence variations in *psb*A-*trn*H region among Myrrh species.

However, our BLAST results revealed a significant misalignment, with only 8% of the myrrh samples matching the species specified in the Pharmacopoeia of the People’s Republic of China. Most of the samples, in contrast to the guidelines, belonged to different species within the genus *Commiphora*, indicating a wider genetic diversity. Particularly striking was the identification of *Liquidambar formosana*, whose sequences were clearly distinguished in the BLAST comparison, confirming its identity. This divergence underscores the efficacy of our genetic approach in accurately identifying both genuine myrrh species and potential adulterants, offering a comprehensive view of the genetic landscape. For a visual representation of these findings, refer to Figs. 3 and 4.

### Analysis of inter- and intraspecific distances for myrrh and hybrids

**Fig 5:**
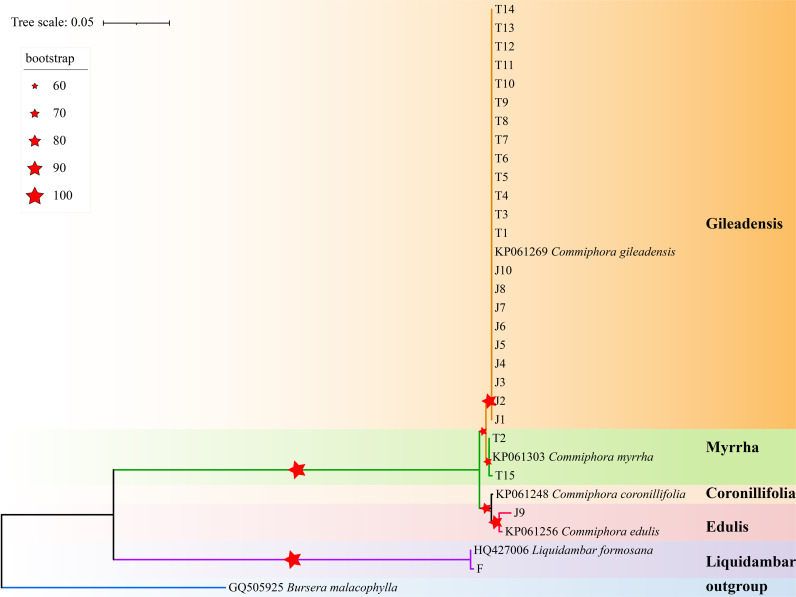
Phylogenetic analysis of *Commiphora* Species and *Liquidambar formosana* based on *psb*A-*trn*H Barcode sequences.

Table 3 presents a detailed genetic analysis of 25 myrrh sequences, revealing the presence of three distinct species: *Commiphora gileadensis*, *Commiphora myrrha*, and *Commiphora edulis*. These sequences, analyzed through the *psb*A-*trn*H region with an average G-C content of 29.7%, spanned a compact spacer region of 427-437 bp. Despite having only 15 out of 438 variant sites across four haplotypes, the *psb*A-*trn*H sequences effectively differentiated between the three myrrh species. Fig 5 illustrates the genetic differences among the haplotypes. *Commiphora gileadensis* showed concordance at specific loci with variations at loci 205, 306, and 388. *Commiphora myrrha*, represented by two haplotypes, exhibited a unique mutation at locus 58. Whereas *Commiphora edulis* is inconsistent with all the other three haplotypes at these 15 loci. Additionally, the genetic analysis included *Liquidambar formosana*, revealing 88% *Commiphora gileadensis*, 8% *Commiphora myrrha*, and 4% *Commiphora edulis*, contrasting with the Pharmacopoeia of the People’s Republic of China specifications that emphasize *Commiphora myrrha* or *Commiphora molmol*.

Table 5 displays the intra- and inter-specific K2P genetic distances for myrrh and its hybrids. The average intraspecific distances were lower than the interspecific distances, demonstrating the gene’s ability to distinguish *Commiphora* species and potential adulterants. Notably, the interspecific distances with *Liquidambar formosana* ranged from 0.6917 to 0.7094, which is much greater than the genetic distance between *Commiphora* genera. All these confirm the effectiveness of *psb*A-*trn*H in identifying *Commiphora* species and hybrids. This comprehensive genetic analysis highlights the diversity within myrrh species and establishes *psb*A-*trn*H as a valuable tool for ensuring authenticity. It suggests the need for updated guidelines and regulations in the herbal markets to recognize and authenticate myrrh more accurately.

**Table 5:** Intraspecific and interspecific K2P genetic distances for *psb*A-*trn*H.

Species	*psb*A-*trn*H			
	Average Length(bp)	Average GC content(%)	Average Intraspecific distance	Average Interspecific distance
*Commiphora gileadensis*	427	29.5	/	0.0223
*Commiphora myrrha*	434	29.5	0.0023	0.0192
*Commiphora edulis*	437	28.8	/	0.0360

### Analysis of Neighbor‑joining tree

In the genetic analysis, three myrrh sequences, one from *Liquidambar formosana*, and the outlier *Bursera malacophylla*, were included in a cluster analysis. The phylogenetic tree based on the *psb*A-*trn*H region, constructed using MEGA-X, reveals branches with robust support, exceeding 50%. Fig 5 illustrates this distinct clustering. Sequences from *Liquidambar formosana* did not cluster with those from the genus *Commiphora*. Instead, the 25 myrrh sequences are divided into three distinct species: *Commiphora myrrha* (two sequences), *Commiphora gileadensis* (22 sequences), and *Commiphora edulis* (one sequence). The *psb*A-*trn*H region effectively distinguishes between *Commiphora gileadensis*, *Commiphora myrrha*, *Commiphora edulis*, and the potential outlier, *Liquidambar formosana*. This phylogenetic tree emphasizes the reliability of the *psb*A-*trn*H region as a barcoding tool for verifying the authenticity of myrrh.

## Discussion

In the “Myrrh Properties” of Pharmacopoeia of the People’s Republic of China, Gum myrrha and Gum opoponax are similar. Although there are minor morphological differences between myrrha and opoponax, which the naked eye can distinguish, it is not possible to distinguish the species. In the ever-evolving intersection of modern science and traditional herbal medicine, the exploration of Chinese herbal medicines has become a focal point for scholars worldwide [6]. The genetic odyssey undertaken in this study delves into the intricate landscape of myrrh species, presenting a Pandora’s box of discussions that resonate across phylogenetic intricacies, practical applications, and broader implications for both herbal markets and medicinal plant research. The selection of the *psb*A-*trn*H region [10,31,32] as the linchpin of this genetic exploration emerges as a strategic choice. This genomic compass unravels the tapestry of genetic diversity within myrrh species with commendable efficiency. Its ability to amplify consistently and discriminate effectively underscores its pivotal role in phylogenetic analyses [11]. This aligns seamlessly with previous studies advocating for the use of this chloroplast region to resolve taxonomic relationships across diverse plant groups. The unwavering identification of key myrrh species—*Commiphora gileadensis*, *Commiphora myrrha*, and *Commiphora edulis*—coupled with the adept detection of counterfeits like *Liquidambar formosana*, serves as a testament to the robustness of *psb*A-*trn*H. This echoes the chorus of prior research, reinforcing the suitability of this marker for delineating taxonomic intricacies. The immediate implications of these findings reverberate through the herbal market landscape, raising legitimate concerns about the authenticity of myrrh-based products. The prevalence of *Commiphora gileadensis*, diverging from the Pharmacopoeia of the People’s Republic of China-specified *Commiphora myrrha* and *Commiphora molmol*, calls for rigorous quality control measures. Regulatory bodies and manufacturers must heed these genetic insights to ensure precise product labelling, fostering consumer trust in the authenticity of myrrh products [2].

The research unfurls the challenges associated with pharmacopoeial specifications, with only 8% alignment of myrrh samples with Pharmacopoeia of the People’s Republic of China-specified species. This underscores the necessity for continuous reassessment and adaptive updates in pharmacopoeial standards to mirror the dynamic nature of plant genetics. Beyond the pragmatic realms of herbal markets, this study makes a resonant contribution to the broader domain of medico-botanical research. Accurate species identification emerges as the cornerstone for unravelling the medicinal properties of myrrh. The unique genetic profiles spotlighted by *psb*A-*trn*H not only authenticate myrrh samples but also lay a foundation for exploring therapeutic variations within distinct species. This discourse paves the way for deeper investigations into the medicinal attributes linked with specific myrrh species, potentially guiding more targeted and efficacious herbal interventions. Looking ahead, the study serves as a prologue to future research avenues across various fronts. In the phylogenetic realm, exploring additional chloroplast regions and nuclear markers could amplify the resolution of genetic relationships. When it comes to myrrh authentication, the integration of molecular tools like DNA barcoding into routine herbal product testing could revolutionize the industry. Collaborative efforts among researchers, regulatory bodies, and industry stakeholders stand as a linchpin for crafting comprehensive and adaptive pharmacopoeial standards, resonating with the dynamic landscape of botanical discoveries. Moreover, our genetic exploration opens avenues for future research, both in understanding the therapeutic variations within distinct myrrh species and in advancing the integration of molecular tools like DNA barcoding into routine herbal product testing. Collaborative efforts among researchers, regulatory bodies, and industry stakeholders are pivotal for shaping adaptive pharmacopoeial standards that resonate with the evolving landscape of botanical discoveries. In essence, our study acts as a prologue to a deeper understanding of myrrh, bridging the gap between its historical significance and its genomic intricacies in the modern era.

## Conclusion

The investigation utilizing the *psb*A-*trn*H region has proven its effectiveness as a reliable genetic marker for distinguishing myrrh species and detecting potential counterfeits. It successfully differentiated *Commiphora gileadensis*, *Commiphora myrrha*, and *Commiphora edulis*, while also identifying *Liquidambar formosana* as a potential adulterant due to its distinct genetic profile. These findings have significant implications for the herbal market, underscoring the need for stricter quality control measures and accurate product labeling. Beyond market considerations, precise species identification forms the basis for understanding therapeutic variations among myrrh species, potentially guiding more targeted herbal interventions. This highlights the need for future research into additional genetic markers to improve resolution and calls for the adaptation of pharmacopoeial standards to reflect the evolving landscape of plant genetics.

## Collection and use of samples statements

The collection and use of plant material in this study were conducted in strict accordance with all relevant national, international, legislative, and institutional guidelines
